# The Personal and Professional Development Experiences of Paediatric Nurses: An Interpretative Phenomenological Analysis

**DOI:** 10.1155/jonm/4185253

**Published:** 2026-09-07

**Authors:** Leah Rosengarten, Laura Jillian Park, Marco Tomietto

**Affiliations:** ^1^ Department of Nursing, Midwifery and Health, Faculty of Health and Life Sciences, Northumbria University, Newcastle upon Tyne, UK, northumbria.ac.uk

## Abstract

**Aim:**

To explore paediatric nurses’ experiences and perceptions of personal and professional development (PPD).

**Background:**

PPD is a crucial factor in enhancing nurses’ job satisfaction and retention. Whilst this topic has been widely explored in general nursing, limited research exists on how paediatric nurses perceive and experience PPD. Registered nurses at the standard clinical grade (Band 5) comprise the majority of the children’s nursing workforce, but are rarely the focus of PPD.

**Design:**

This UK‐based qualitative study used interpretative phenomenological analysis (IPA) following COREQ guidelines. Trustworthiness was ensured through researcher reflexivity, PhD supervision and member checking.

**Methods:**

A purposeful sample of 15 nurses working in acute paediatric services across four organisations were interviewed, with a range of ages, experiences and work qualifications. Each participant was interviewed twice, and data were analysed using a seven‐step IPA framework.

**Results:**

Four overarching themes emerged: Individual impact, work location, employing organisation and external factors. PPD was experienced as a dynamic, non‐linear process shaped by intrinsic motivation, personal circumstance, workplace culture and managerial gatekeeping, staffing constraints, organisational funding and geographic inequity. Paediatric nurses felt undervalued when compared to other nursing fields, experiencing limited field‐specific development.

**Conclusions:**

This study suggests the implementation of organisational strategies at the unit and hospital level to support PPD. To shape the most effective approaches, it is also necessary to consider individual and extra‐organisational levels, which might affect the human resource management in this area.

**Implications for Nursing Management:**

Nurse managers must act as enablers rather than gatekeepers of PPD. It is key to ensure equitable access to opportunities, with adequate staffing and field‐specific pathways of development. National minimum standards for paediatric nursing PPD are needed to address systemic inequity.

**Highlights**
• Personal and professional development for paediatric nurses is unique.
• Individual, work location, organisational and external factors all impact on PPD.
• Organisations should consider institutional pathways for recognising personal and professional development.

## 1. Introduction

Career development in nursing involves skill advancement, education and professional growth, which can take different forms. Entry level nurses are significantly associated with high levels of turnover [1], and the lack of personal and professional development (PPD) opportunities in the nursing profession is one relevant component of the intention to leave the profession [2, 3].

The term PPD is often used interchangeably with the term ‘Continuing Professional Development’ (CPD) and includes concepts such as personal development and career progression. For this study, PPD was the term used, which refers to ‘the individual’s own learning and experiences which enable the advancement of personal and professional knowledge and skills, growth of personal capabilities and career expansion’ [4]. Whilst CPD is a commonly used term in healthcare, it is inconsistently defined and understood, meaning that this concept means different things in different contexts [5]. CPD in healthcare literature is often seen to focus purely on the educational aspects of development with a disregard for the personal aspects [6, 7]. Professional development is a broader term often encompassing all development [8] where personal development focuses solely on the individual. Workforce development, career development and continuing professional education are further terms that are also used interchangeably in the literature.

The benefits of PPD for nurses are well documented: it is argued that PPD creates a skilled, knowledgeable and compassionate workforce [9]. The World Health Organization’s (WHO) Global Strategic Directions for Nursing and Midwifery 2021–2025 [10] note four different priorities: education, jobs, leadership and service delivery. Each of these priorities links to PPD, as the continuous evolution of healthcare requires the evolution of both undergraduate healthcare education, and professional knowledge and competencies of the existing workforce. Within each of these domains, there is a focus on helping nurses to develop to meet global health priorities, to recruit and retain nurses, to increase leadership skills and to enable nurses to maximally contribute to service delivery. The global focus on the need for PPD for nurses is also emphasised in target 3c of the United Nations’ 2030 Agenda for Sustainable Development [11] and in the International Council of Nurses [12] work to advance the nursing profession.

Whilst there is discussion in the literature on the difficulties of evidencing the effectiveness of PPD [13], much literature argues that PPD is essential for improved patient outcomes and enhanced quality of care [7, 14, 15]. Furthermore, PPD has positive consequences for the organisation, not just through improved patient outcomes but also through increased job satisfaction and therefore staff retention [15, 16]. Kasdorf and Kayaalp [2] demonstrated that employee perceptions of development are significantly related to the intent to stay and job satisfaction.

The literature shows that PPD strategies for nurses have traditionally been focused on fields that have problems with retention or recruitment, leading to low or declining staffing numbers such as learning disability and primary care nursing [17]. As such, paediatric nursing PPD often gets little focus. Available studies that consider paediatric nursing PPD are often dated and have limited transferability as they are based in countries where nurses are not educated to practice in specific disciplinary fields at the undergraduate level [18], they focus on newly qualified staff who have experienced limited post‐registration development [19] or they focus on staff who have already experienced career progression to advanced nursing levels [20].

The focus on PPD is increasingly important in the context of the ageing nursing workforce, a challenge documented across international contexts [21]. As experienced nurses approach retirement and the paediatric nursing workforce becomes proportionately more junior, there is a need to invest further to develop this cohort and keep the skill levels and quality of care patients require. At the same time, rising global demand for paediatric services and an increase in patient acuity mean that paediatric nurses must develop to manage the increasing demands placed upon them by patient need [22–24]. As much of the existing literature on nursing PPD is presented from a general nursing perspective, this study is both timely and pertinent to shed light on the needs of the paediatric nursing workforce.

Focusing on paediatric nursing as a career, most countries such as the USA [25] and Australia [26] choose to educate nurses in generalist programmes before nurses can opt to complete post‐graduate qualifications in their chosen specialism. Differing from this, in the United Kingdom (UK), nurses are educated at the undergraduate level in one of four fields of nursing; adult nursing, children’s nursing, learning disability nursing or mental health nursing. Germany and the Republic of Ireland are two countries known to also educate undergraduate nurses in field‐specific nurse education programmes [27, 28]. As UK paediatric nurses specialise from the point of undergraduate education, evidence focusing on PPD in other countries may not directly transfer to the UK context. This does not distract, however, from the importance of PPD in paediatric nursing as it is known that PPD contributes to improved competence, increased job satisfaction and mitigates burnout for the paediatric workforce [29, 30]. Understanding how paediatric nurses experience PPD within this distinctive context is therefore key and provides the rationale for this study.

## 2. Methods

### 2.1. Aim

The aim of this study was to explore Band 5 (registered nurses at the standard clinical grade) paediatric nurses’ experiences and perceptions of PPD.

### 2.2. Study Design and Theoretical Framework

This study employed a qualitative design, following the COREQ guidelines [31]. The philosophical underpinnings of this study propose that individuals may perceive PPD differently depending on their personal views, experiences, and demographics, influenced both by the meaning they have attributed to the phenomenon and through various experiences and interactions with this concept throughout their career. As such, interpretive phenomenological analysis (IPA) was the methodology chosen for this study as it allowed for interpretation of individual’s experiences and a unique, in‐depth analysis [32].

IPA is a methodology that Smith [32] identified as a ‘double hermeneutic’ phenomenology, which is useful for the examination of subjects that are complex, ambiguous or particularly emotive. IPA is known as a double hermeneutic as ‘the researcher is trying to make sense of the participants trying to make sense of their world’ [33]. There are three key underpinnings to the IPA approach, phenomenology, hermeneutics and ideography [34]. These underpinnings involve an interest in lived experience, a belief that interpretation is intrinsic and a focus on detail and depth of analysis [34].

Utilising IPA as a methodology, this study employed semistructured interviews to explore Band 5 paediatric nurses’ experiences and perceptions of PPD. The interviews explored the participants lived experiences of PPD, considering the meaning and value that they placed on this phenomenon. The full interview guide is listed in Table 1. This guide provided broad, open prompts with questions focussed on how PPD is enhanced or restricted and how their experiences could be better supported in future.

**TABLE 1:** Interview guide.

1. What does personal and professional development mean to you as a Paediatric Nurse? Why?
2. Is development important to you and why?
3. Can you please tell me about your experiences of personal and professional development as a Paediatric Nurse so far?
4. What do you feel supports your personal and professional development as a Paediatric Nurse? Why?
5. What do you feel hinders your personal and professional development as a Paediatric Nurse? Why?
6. How do you think personal and professional development could be improved?
7. Is there anything personal to you, within any stages of your life that you think may have had an impact on your development?
8. Is there anything else you would like to add about personal and professional development?

This study is based in the UK where the National Health Service (NHS) nursing roles are structured by pay band, with Band 5 being the first pay band for a registered nurse role. Band 5 nurses deliver much of the direct clinical care to patients, and this pay band reflects role and pay, not experience or seniority. Nurses who progress up the pay bands will take on increasing responsibilities at Bands 6–8, usually ward sister or charge nurse, ward manager and then matron positions. Band 5 paediatric nurses comprise most of the paediatric nursing workforce but are often not the focus for PPD studies, hence the area of interest within this study.

### 2.3. Rigour and Trustworthiness

Lincoln and Guba [35] argue that trustworthiness of qualitative findings is enhanced through researcher reflexivity. Within this study, a research diary supported note taking to allow for frequent reflexivity through each stage of the study, whilst frequent PhD supervision enabled discussion and reflection with the wider research team. To further strengthen trustworthiness, member checking was undertaken, whereby participants were invited to review and verify the accuracy of the findings, ensuring that the interpretations authentically represented their experiences [36]. Two weeks minimum were left between interviews to allow participants time to reflect on the first interview.

Consistent with an IPA approach, bracketing was understood to be a focus on ongoing reflexive acknowledgement and suspension of preconceptions, rather than a single event [34]. As the lead researcher, LR is a registered paediatric nurse with experience of the phenomenon. As such, prior to commencement of the research, LR documented any assumptions and preconceptions in the research diary. These were then revisited throughout the study. This process supported open, non‐leading questions during the interview, and supervisory discussions were utilised to identify where LR’s own experiences might shape interpretation. This ensured that results remained grounded in participant’s accounts.

### 2.4. Sampling and Recruitment

A purposeful sample of paediatric nurses was recruited. Inclusion criteria for the study required that participants were registered paediatric nurses working at Band 5 level in the NHS, with more than 1 year post qualifying experience. The exclusion criteria excluded paediatric nurses who are employed at advanced nursing levels, and those who did not work in acute hospital settings. This exclusion criteria were chosen as to include other groups that were felt to create too many varying factors for the scope of this study.

Once participants had volunteered, a phenomenal variation [37] was applied to the sample, using a sampling matrix, to select participants from varied units and hospitals, of different ages, with varied levels of experience and varying qualification levels. The homogeneous sample required for IPA research was created through shared experience of the phenomenon of PPD. Including diversity within the sample ensured variation within the phenomenon and allows increased understanding of how diverse factors shape experiences, thus increasing the transferability of the data gathered [37].

### 2.5. Data Collection

Potential participants were approached by email, delivered to their secure NHS email account. Within each NHS organisation employing these staff, a ‘gatekeeper’ was identified who was asked to share the recruitment email with appropriate staff. Gatekeepers were nurses responsible for education or management of paediatric nurses within each Trust, identified either through the lead researchers’ own contacts or the Trust R&D departments. The research was also advertised via social media, asking for voluntary participants. From this recruitment, 27 participants volunteered to participate in the study. Twelve volunteers were excluded as they did not respond to interview scheduling requests or had demographics already represented in the sample. They were thanked for their interest.

Face‐to‐face, individual semi‐structured interviews were initially planned; however, due to government‐sanctioned COVID‐19 pandemic restrictions, interviews took place online via zoom (supported by ethics). Participants were interviewed twice by LR to verify the accuracy of the findings and allow for exploration or clarification of developing themes [36]. Interviews were chosen as a method as they afford the opportunity to obtain detailed accounts from participants, which allowed for exploration, understanding and communication of the participant’s experiences and viewpoints [38]. Interviews were audio‐recorded by Dictaphone and supported by an interview guide (Table 1), whilst the researcher also took notes during the interviews to support later analysis [39]. Transcription was conducted by LR, aided by Microsoft Word’s automated transcription. Interviews took place between 12/03/2021–6/4/2021 and 5/4/2022–17/5/2022 due to LR having time off on maternity leave.

In total, the sample population included 15 participants from paediatric services within four acute NHS healthcare organisations in the Northeast of England. Interviews ranged from between 13 and 41 min in length but averaged at 25 min in length. The length of interviews and depth of discussion were guided by participant responses.

### 2.6. Data Analysis

Data were analysed using the seven steps of IPA identified by Smith et al. [40]. Following the first interviews, the data were inductively analysed by LR, before inviting feedback about the analysis from LP and MT, and then the participants in a second interview, with the view to increase the credibility of the data and empower the participants [36].

Data were initially transcribed and read by the researcher to allow for familiarisation with the data and initial noting, with transcripts read multiple times [1]. Inductive notes were written by hand to capture initial impressions and a holistic sense of each participant [2]. Following this, experiential statements were constructed, demonstrating key concepts within the data [3]. Next, analysis involved searching for connections across the experiential statements, to integrate statements into emergent themes [4]. The above stages were repeated for each of the interviews [5], with initial statements reviewed iteratively to ensure they remained grounded in participant accounts rather than being shaped by preconceived ideas from the literature or the authors’ own experiences. The transcripts were then inputted into NVivo Qualitative Data Analysis Software [41] to support data organisation, transparency and auditability. Following this, patterns across cases were identified and NVivo’s [41] query functions were used to explore relationships, check category consistency, and consider convergence and divergence in the sample [6]. Finally, through repeated checking and refinement, the emergent clustered into four overarching themes to allow for the writing up of the analysis [7].

### 2.7. Ethical Considerations

As this study involved recruitment of NHS staff through their workplace, it required Health Regulatory Authority (HRA) approval alongside academic ethical approval. (Institution blanked out) ethical approval was granted on 12 May 2020 (ref. 23,450), and HRA ethical approval was granted for this project on 10 July 2020 (ref. 282,366). Participants gave informed written consent by email prior to the interviews and were advised that they were free to withdraw from the study at any time. They were also able to withdraw their data from the study up until data analysis had taken place. Data were handled according to General Data Protection Regulations [42].

## 3. Findings

Fifteen participants were recruited for this study. One participant completed the first interview but, after relocation, did not respond to invitations for a second interview. Demographics of the participants are displayed in Table 2. Participant’s responses were anonymised using participant identification numbers.

**TABLE 2:** Demographic information of study participants.

Participant	Age	Years experience	Gender	Qualification	Work location
1	24	3	Female	Bachelor’s Degree	Specialist Hospital 1
3	24	2	Female	Bachelor’s Degree	Specialist Hospital 1
4	27	3	Female	Bachelor’s Degree	Specialist Hospital 1
5	27	7	Female	Bachelor’s Degree	Specialist Hospital 1
7	48	27	Female	Diploma Higher Education	Specialist Hospital 1
8	54	13	Female	Master’s Degree	Specialist Hospital 1
11	24	2	Female	Bachelor’s Degree	Specialist Hospital 1
12	31	6	Female	Bachelor’s Degree	Specialist Hospital 4
15	31	5	Female	Post Graduate Certificate	Specialist Hospital 2
17	24	1	Female	Bachelor’s Degree	Specialist Hospital 1
20	34	8	Female	Master’s Degree	Specialist Hospital 1
22	32	9	Female	Bachelor’s Degree	Specialist Hospital 3
25	30	8	Female	Bachelor’s Degree	District General 1
26	32	10	Female	Bachelor’s Degree	District General 1
27	43	5	Female	Bachelor’s Degree	District General 2

Four overarching themes emerged from the data: Individual impact, work location, employing organisation and external factors. These will be presented below and discussed in the next section.

### 3.1. Individual Impact

This theme captures the internal factors shaping how Band 5 paediatric nurses make sense of their development. Participants described PPD as an intrinsic part of both their day‐to‐day role and their long‐term career path. Participants consistently described the way that individual factors impacted on their experiences of PPD. For many, their own perception of PPD was that it was essential to keep ‘up to date’ or to deliver competent patient care. It was clearly felt that a nurse could not be fully effective in their role, if they did not participate in PPD.

The participants’ goal from participating in PPD showed variance, however. For some, PPD was about gaining confidence in their skills and abilities as a nurse, where for others PPD was about progressing up the career ladder

‘Like a lot of people are like quite happy being a band 5 forever… other people really want to progress and see it more as a status’ (Participant 20, interview 1).

It was clear in the reporting of PPD experiences that a participant’s personality played a role in their experiences of PPD. Often, participants identified that the reason they felt they had developed was because they were motivated to participate in PPD. Participants often compared their own motivations to those of their peers, feeling that intrinsic motivation was the most important factor behind a successful PPD experience, as highlighted below:

‘Like it′s a lot of it comes down to the individual’ (Participant 27, interview 1).

The chance to participate in PPD was frequently seen as an exciting benefit to working as a nurse, and a recurring tension from participants across different hospitals arose when reporting occasions in their career where there had been bias around development opportunities. This produced feelings of unfairness, particularly when decisions appeared predetermined, rather than based on merit:

‘There was a few of us, put our names forward, but then it was obvious they already had one person picked out for it and then it was just never mentioned again… It was as if like it was who you knew rather than what you knew rather like to get into it’ (Participant 22, interview 1).

Another emotive subject that participants struggled with, was the impact of their personal life on their career. Nurses who were older and had more years of experience discussed this at more length, largely due to the increased number of experiences they had to reflect upon. Participants reported a conflict between wanting to develop in their career, whilst also experiencing occasions when they lacked the time, energy or motivation to engage in PPD. Having children was often listed as a barrier to PPD, whether because this necessitated part time working, because this reduced their ability to be flexible in working hours, or because participants felt they lacked the energy needed to seek out or engage in PPD. Other key life events such as bereavement and relationship breakdowns were also recognised as having monumental impact on a nurse’s career:

‘There might be an opportunity to come up for progression and further learning in the role I′m in, but I’ve got a relatively young child and yeah, being able to have that, am I going to have the right time, the amount of time? Availability to to do it with my life at home’ (Participant 12, interview 2).

There was often a lack of awareness of opportunities for PPD, either through participants being unaware that opportunities existed, or not knowing how to access these. Whilst participants also felt that their PPD should be funded by their employer and happen during work time. On occasions that PPD was either paid for by the individual or done in their own time, there was a sense that this constituted going ‘above and beyond’ what should be expected:

‘I would probably to be honest, I would probably pay for courses if I thought it was going to benefit what I was looking to do, but I know other people maybe wouldn’t be in the position to do that’ (Participant 1, interview 2).

Overall, this individual impact theme highlights that PPD is first interpreted individually before nurses engage with external impacts that either challenge or reinforce their PPD.

### 3.2. Work Location

The ‘Work Location’ theme demonstrates how the individual impact above, can be either strengthened or challenged by the participants’ workplace environment. The culture, structure and resourcing of their immediate clinical area played a significant role in whether PPD felt supported, constrained or blocked completely.

A key part of this experience was the variation between units, even within the same organisation. Units which were seen as supportive to PPD were considered a deciding factor for the unit a nurse chooses to work on, and a lack of PPD was cited as a reason for intent to leave:

‘Where I work particular (named unit) I, the reason I chose it was cause I knew there was going to be a lot of professional development like handed to me or like not on a plate. But I knew that I would learn lots from the day one’ (Participant 15, interview 1).

Workplace culture, particularly the behaviour of peers and managers, was described as central to experiences. For nurses who were younger or newer to the profession, the support they received from their colleagues was pivotal. If colleagues were seen to be supportive, participants felt they could ask questions and be open about their development needs, where unapproachable colleagues left participants unwilling to seek support:

‘Cause you know which certain people you would rather go and ask than other people. Because, you know? There’s some people that will help you with anything and some other people that might be a bit more hesitant to’ (Participant 11, interview 2).

Managers were framed as gatekeepers to PPD, having the power to approve training, release staff from duties and authorise funding. Below, participant 26 reflected on working under a manager who was felt to be stingy in allowing nurses to access PPD funding:

‘I think sometimes (name of manager) can hold people back a little bit cause I remember wanting to do my clinical skills^1^ on the ward as well. And her saying well, there’s a list of people that want to do it’ (Participant 26, interview 1).

Where Clinical Educators were mentioned within the research, they were universally positively perceived. Participants valued their skills in identifying opportunities for PPD, access these and delivering some PPD opportunities themselves. One specialist hospital had recently employed its’ first Clinical Educator in Paediatrics shortly before the research interviews took place. Both participants who worked in this location offered multiple examples of the benefits they had perceived for their PPD since having the Clinical Educator in post:

‘She’s very passionate. Like if I said ooh I want to develop this way. She’d say right, get you on that study day, do this. Go round and shadow these people’ (Participant 11, interview 1).

Staffing levels, equipment, funding and time were a persistent barrier to PPD. The ability to access computers to complete mandatory training was a common problem. Participants described occasions when training could only be undertaken outside of working hours or if paid for by the individual:

‘She wanted to go for a secondment to another job and then they told her that she could only do it if she like did it as like additional like overtime to her job. So, she works like her 30 hours and then she does another day like with this secondment’ (Participant 20, Interview 2).

These examples reinforced the tension experienced when participants wanted to develop but felt practically unable to do so.

### 3.3. Employing Organisation

Beyond their immediate work environments, participants situated their experiences in the wider organisational landscape. They described variation in an individual’s experiences of PPD according to the organisation they worked in, in terms of opportunity, culture and funding. It was assumed that the larger the organisation, and the more paediatric nurses the organisation employs, the better the opportunities for PPD would be. Yet, participants who had worked, or were working in larger organisations, reported this was not always the case:

‘I feel like from working in different trusts … they do put on like a lot of courses and things like you’ve got a lot of good like clinical educators are really good and they seem to have a lot of training opportunities’ (Participant 20, interview 1).

Financial and structural barriers were seen to be shaped at organisation or NHS level. Pay was also cited as a barrier to PPD within the NHS, as participants reported that where they were expected to take on advanced nursing roles or specialist skills, with no recognition of this in their pay grade, they often avoided participating in the PPD:

‘I′m expected as part of my appraisal now to be trained on this machine, (name removed) and obviously a (job removed) gets paid (£50–60,000). Well beyond what our wages would ever meet’ (Participant 4, Interview 1).

Revalidation is the process of nurses in the UK demonstrating their continued ability to practise safely and effectively, therefore maintaining registration with the Nursing and Midwifery Council [43]. Although revalidation did not feature prominently for all participants, those who discussed it reported revalidation as a helpful process, which supported them to reflect on their development. A few expressed being anxious about meeting hour requirements due to the constraints discussed above, demonstrating how individual motivation interacts with organisational conditions.

### 3.4. External Factors

Participants also located their experiences of PPD within the wider context outside of the organisation. COVID‐19 was identified as having a significant influence on development, both negative, due to a reduction in PPD opportunities, and positive, due to the opportunity to develop new skills. This reflects the complexity of PPD at a time of crisis, as development was necessary, but not always chosen.

Geography was a prominent factor, exemplified by a perceived North–South divide, with PPD seen as more readily available in London than in the Northeast. Participants were discontented by the knowledge that nurses they knew working in other areas were promoted faster and paid more for doing the same job role than nurses in the Northeast:

‘Like and she’s now got a band 7 at the same time as I got my 6, but everything that she’s doing as a 7, it’s the same as the stuff that I′m doing is a six’ (participant 26, Interview 2).

Further discontent was displayed when participants reflected on the differences between paediatric nursing and other nursing specialities. Being a paediatric nurse was cited as barrier to PPD as it was felt that there were more opportunities for PPD and promotion in the other fields of nursing. There was a strong sense of pride amongst the participants that paediatric nurses were required to have specialist knowledge and skills that set them apart from other fields of nursing, yet they felt these qualities were not recognised by others:

‘I think band five children nurses are maybe, not undervalued, but well, yeah, maybe undervalued, I think’ (Participant 15, interview 1).

Wider reflections on nursing as a career brought these reflections on external influences together. Participants identified that nursing was lacking the linear progression, in terms of both pay rises and job roles, that other career paths offered. Frustration was expressed that the effort individuals feel they put into their PPD and the complexity and difficulties of their job role are not reflected in their pay:

‘You′ve developed yourself and you’ve done all of these study days, but then you’re still coming in and doing the same job that you were doing however many years ago. For the same pay’ (Participant 26, Interview 2).

### 3.5. A Timeline of PPD

A timeline of PPD became apparent through asking participants to reflect on their careers up to the point of interview. Participants disclosed how preceptorship was a time of structured development with a lot of support. Early in their careers, nurses seemed to focus on developing personal skills such as resilience and confidence. Throughout the next few years, PPD was centred around skills needed to perform the job role. Following this, participants appeared to be interested in PPD which would help them to progress to advanced nursing roles. For those participants much further in their career, PPD seemed to be separated into that which they had to do to retain competence in their role, and that which they chose to do because it was of specific interest to them.

## 4. Discussion

This study aimed to explore Band 5 paediatric nurses’ experiences and perceptions of PPD. Together, the themes presented illustrate how Band 5 paediatric nurses negotiate their development within a complex interplay of personal factors, workplace environment, employing organisation and wider social context. PPD was not described as a linear process, but as a dynamic thread present throughout their careers, shaped by the interaction of each of the above themes.

Intrinsic motivation is a key individual factor for PPD [44–46]. Horn et al.’s [47] study of paediatric nurses’ development highlighted that the highest‐ranked motivators for professional development were because individuals desired an increased knowledge base to improve patient care, personal fulfilment or for an increased clinical role. This is consistent with both self‐determination theory and an interactionist perspective about career development: whilst the organisation should provide opportunities for development, the individuals should also demonstrate intrinsic motivation and proactive behaviours to develop themselves [48, 49].

This study also highlighted that family commitments can be a barrier to PPD. This is supported by Horn et al. [47], Mao et al. [50], Yu et al., [51] and Hakvoort et al. [52], whose studies all report that family commitments can act as a barrier to participating in PPD due to a lack of time or flexibility. Moreover, work–family balance in nursing is extensively recognised as a key factor to enhance job satisfaction and retention [53]. Considering individual barriers further, Palma et al. [54] and Hakvoort et al’s [52] studies both focused on barriers to development for nurses and found that an individual’s time and ability to fund PPD were some of the biggest challenges faced by nurses, though neither of these studies examined the views of paediatric nurses as a distinct group.

In this study, individuals chose where they would like to work according to the work location and their plans for career development. This is supported by Tiliander et al. [55] who sought to understand nursing students’ motivations for choosing certain specialities and found that a desire to achieve personal development was a key motivator for speciality choice. This is interesting to consider when contextualised against the fact that paediatric nursing is often considered a popular speciality of nursing [56, 57]. It is therefore curious to consider that participants in this study reported comparatively limited opportunities for development and promotion, despite the speciality’s popularity. This may, in part, reflect the limited attention that paediatric PPD has received in the research literature. As this concern has not been well studied, it is not clear whether this affects only a minority of paediatric nurses or represents a more widespread but hidden inequity.

The way that paediatric nurses experience development over their careers is supported by the wider literature from other fields of nursing, which identifies that age or length of time qualified has an impact on the way nurses experience or perceive PPD [5, 44, 54, 58–60]. Participants in this study who were newer to the profession reported an increased value on the support of colleagues. The impact of age and the suggestion that younger workers receive more schooling and on‐the‐job training than older workers do is also established in the seminal work on human capital theory by Becker [61], so this bears consideration for the development of future PPD opportunities. This leads onto the question of how senior nurses in advancing nursing roles may experience their PPD. As this study focuses on the development of Band 5 nurses, the experiences of senior colleagues in Bands 6 plus has not been established and represents a gap in the wider literature.

The impact of the culture of a workplace on PPD is widely supported in the literature on paediatric nurses, with studies by Rosengarten and Callum [45] and Taylor and Foster [46] reporting the importance of a supportive workplace. This extends beyond support from managers in Darvill et al.’s [62], Hu et al.’s [63] and Buckley et al.’s [30] studies where the role of the peers in providing support is also established. The varied cultures across units experienced by participants in this study contradict the participants’ own beliefs that certain employing organisations are more supportive than others, as contrasting cultures were reported within individual organisations. Participants employed in areas where there was a dedicated clinical educator reported clear benefits for their PPD. This suggests that specialist infrastructure may shape opportunity more than organisational size.

Interestingly, the role of the clinical educator in supporting paediatric nursing PPD is not widely discussed in the paediatric nursing literature, though Coventry and Russell [64] identified that graduate nurses felt that the ‘clinical sympathy’ they were offered from clinical educators was essential to their role and development. This is an important concept that has arisen from this study, which requires further research.

This study identified that a lack of staffing acts as a barrier to PPD and global nursing shortages undoubtedly impact on this. This finding is well supported in the literature, with research indicating that where staffing shortages exist, resources must cover the essential provision of care, thus limiting opportunities for PPD [6, 65–67].

The level of support an employee perceives they receive from their employer is important and is well established both in seminal workforce literature [61, 68, 69] and in healthcare‐focused research in recent years [70]. It is additionally affirmed that there is a perceived lack of financial support for development within some healthcare organisations [15, 47]. Financial and resourcing issues are one of the major components for the opportunities for PPD, with studies demonstrating how the lack of resources is a global issue for workforce’s retention and development [14, 15, 54].

Linking further to finances, nurses within this study felt that being paid appropriately for their role was important in recognising their skill set. Whilst more is needed to be known on the impact of pay, Göktepe et al.’s [71] study of factors associated with job satisfaction for nurses does identify that satisfaction with salary and satisfaction with job opportunities were both positively associated with nurses’ job motivation. Additionally, Bhatnagar et al. [72] showed that insufficient wages were demotivating for nurses. Within Horn et al.’s [47] study, whilst pay was listed as a motivator for PPD, it was the lowest‐ranked motivator listed in the study. That nurses expect their pay to reflect the complexity of the role the nurse performs, and the level of PPD they have had, is lesser established in the literature and would be an interesting area for further research.

A key finding from participants in this study was that they felt that the needs of paediatric nurses are unique to other fields of nursing. This provides new insight into this group of nurses and, whilst a direct comparison of nursing fields and how they experience PPD is not retrievable within the literature, some comparison of adult nursing specialities is available. Studies have identified that nurses who work in speciality nursing areas find access to PPD easier and have more opportunities for practice development [73]. It has also been identified that nurses experience PPD differently according to the setting that they work in [74] and that educational needs are different across settings [75]. Furthermore, there is evidence that PPD for paediatric nurses is often more difficult to locate than adult nursing CPD, with courses, where they do exist, often cancelled due to low numbers [15].

Participants in this study identified unique concerns with the PPD of paediatric nurses. It was identified that there are limited PPD opportunities, progression is less than in other fields of nursing and that there is a lack of recognition of specialist skills and knowledge. Across each of these concerns, the perception that paediatric nurses are disadvantaged relative to other fields of nursing can be neither confirmed nor refuted by the existing evidence base, as no directly comparable studies exist. This absence is a contribution of the current study, identifying perceived inequities for paediatric nurses that warrant further study.

A new and novel finding from this study was the barriers put in place by a paediatric nurses’ geographical location as this has not been widely discussed in the literature. Of interest, Stoye and Warner’s [76] report for the institute of fiscal studies, which looked at career progression in the NHS, noted that there are regional disparities in terms of career progression, with nurses working in Greater London being 45% more likely to progress than nurses working in the Northeast. This suggests that this experience is found by nurses working in other fields, as well as paediatric nurses.

### 4.1. Implications for Nursing Management

The findings from this study suggest several implications for nursing management. Nurse managers should be supported to act as enablers rather than gatekeepers of PPD, with transparent and equitable processes for approving training, funding of opportunities and release from clinical duties. Given the influence of workplace culture on individual PPD, organisations must foster supportive team environments, protecting time and the resources needed for PPD.

The value that participants placed on dedicated paediatric clinical educators suggests that investment in these roles alongside field‐specific pathways of development are key to ensuring optimal access to opportunities. National minimum standards for paediatric nursing PPD are needed to address systemic inequity. Furthermore, investing in PPD contributes to better patient safety outcomes, a workforce better equipped to adapt to changes in healthcare delivery, and better organisational outcomes [77, 78].

Recommendations for future research are also clear. Further paediatric focused studies are needed to establish whether the inequities perceived by participants are experienced across the wider paediatric nursing population, to compare paediatric PPD to other fields of nursing and to consider variances across different nursing levels. Future work should be designed to explore variances across setting, organisation and a nurse’s career stage. Studies incorporating more diverse samples and nurses from Culturally and Linguistically Diverse (CALD) backgrounds would also strengthen the transferability of this evidence.

### 4.2. Strengths and Limitations

Despite literature suggesting that gender and ethnicity impact on a nurse’s career progression [76], these factors did not come up in this study. This is likely due to the demographic background of participants, as all were white females, as no male or Global Majority participants volunteered for the study, which is a limitation of this study. This lack of diversity may be because the Northeast of England is not a diverse area, with 93% of residents being white and identifying as British or English [79].

The above may alter the way these findings might be transferred to different ethnical groups and limit the transferability of the findings, as the experiences of male and non‐UK paediatric nurses still require further exploration and understanding. Further studies should explore the experiences of PPD of CALD nurses. It is important to note though that most nursing workforces are female, so some transferability is appropriate.

A strength of this study is in the sample size included. Whilst 15 participants may be considered a large sample size for some IPA studies as Smith et al. [34] identify that 3–6 participants are often enough for many IPA studies, IPA studies can often vary in sizes from 1 to 30 participants [80]. The criteria for sample sizes for qualitative studies are often not a specific number, but when data saturation is reached [81].

## 5. Conclusion

This study has established that the experiences and perceptions of paediatric nurses within their PPD are shaped by factors at four levels: within their own self, within their work location, through their employing location or by external sources.

What sets this research apart from other studies is the focus on paediatric nurses working at the standard clinical grade, the population who provide most of the direct care to children and young people. Participants felt that paediatric nurses required opportunities that were tailored to their field of nursing and readily available. As no study to date has explored the differences in PPD between one field of nursing and another, this is an interesting area for future research and an area that future workforce development strategies need to consider.

The findings offered by this study can be utilised by policy makers as a structured lens to design workforce development barriers at each level. The need for PPD opportunities to be field‐specific and the reported inequitable access to PPD could direct inform workforce planning and would support a call for national minimum standards to support PPD access. Finally, the highlighted gap in current policy on field‐specific PPD requirements can be utilised to advocate paediatric nursing to be recognised as a distinct workforce category.

## Funding

This research study was undertaken as part of work towards a doctoral study by Leah Rosengarten. Northumbria University funded this doctoral study and has supported the development of this research.

## Conflicts of Interest

The authors declare no conflicts of interest.

## Endnotes

^1^An additional learning course offered in some areas.

## Data Availability

The data that support the findings of this study are available from the corresponding author upon reasonable request.
